# On the status of transfer in adult third language acquisition of early bilinguals

**DOI:** 10.1371/journal.pone.0247976

**Published:** 2021-03-04

**Authors:** Jorge González Alonso, Eloi Puig-Mayenco, Antonio Fábregas, Adel Chaouch-Orozco, Jason Rothman

**Affiliations:** 1 AcqVA Aurora Center, Department of Language and Culture, UiT The Arctic University of Norway, Tromsø, Norway; 2 School of Education, Communication and Society, King’s College London, London, United Kingdom; 3 School of Psychology and Clinical Language Sciences, University of Reading, Reading, United Kingdom; 4 Centro de Ciencia Cognitiva (C3), Universidad Antonio de Nebrija, Madrid, Spain; Leiden University, NETHERLANDS

## Abstract

The study of linguistic transfer—understood here in terms of the copying of previous linguistic representations—seeks to reveal how domain-relevant prior language knowledge impacts the acquisition and development of new mental representations more generally. Studying sequential multilingualism offers a natural laboratory to observe cognitive-economical mechanisms that avoid redundancy in language learning. One of the key dividing questions between theories of transfer in sequential multilingualism is the extent of transfer, that is, whether a whole previous grammar is transferred (full transfer) or a potentially different source language is selected for each linguistic property (property-by-property transfer). We adopted a novel methodological approach to this question, examining four different linguistic properties from unrelated domains of grammar across the three languages of a heterogeneous population of highly proficient, early Catalan/Spanish bilinguals with different degrees of language dominance and order of acquisition, at the very beginning of (adult) L3 English. Results are variably complex across the different properties, but compatible with a scenario where one of the previous languages, Catalan, was selected as the basis for the initial L3 English grammar of these speakers. We discuss the theoretical implications of these findings.

## 1. Introduction

Among the many variables shaping nonnative language learning, the influence of previously acquired languages has traditionally occupied a central position in the study of sequential (i.e., non-simultaneous) bi- and multilingualism [e.g., 1]. In formal linguistic approaches, the role of the first language (L1) in second language (L2) acquisition became an especially hot topic in the 1990s and early 2000s [e.g., 2–4]. The main driving question was how, and to what extent, the mental linguistic representations of the L1 influenced the acquisition of an L2 by determining the initial conditions of the process (the first version of the L2 grammar). Were L1 representations *transferred* as part of the first L2 grammar? If so, did this happen for reasons of cognitive economy (i.e., to avoid learning from scratch those properties that had already been learned for the L1)? Was it the whole of the L1 that served as the basis for the first L2 grammar [e.g., 5]? Was it only part(s) of the L1 grammar that transferred [e.g., 2, 6]? If so, which types of linguistic knowledge were copied over, and which not?

Two decades ago, these questions were taken up in the context of third or further (L3/L*n*) language acquisition, in light of a very relevant difference with respect to L2 acquisition [see 7]. While, in true L2 acquisition—i.e., for those learners with a single L1—transfer can only come from one source, the L1, there are at least two potential sources of transfer in L3/L*n* acquisition. Moreover, these sources are often in conflict when we consider individual grammatical properties (e.g., one language requires subjects to be overtly realized, while the other allows for phonetically null subjects). Similarly, sometimes they match or approximate the target L3/L*n* property, and sometimes they do not. How does the mind of L3/L*n* learners make use of previously acquired languages to avoid redundancies in learning?

In technical terms, the availability and choice between several previous language representations and their involvement in L3/L*n* acquisition is known as *transfer source selectivity*. Much work has focused on uncovering the underlying mechanisms of this selection [8 for review]. Debates have been articulated around two major questions: (a) what factors condition selection between the L1 and the L2? and (b) does the grammar of one of the previously acquired languages transfer in whole (full transfer, a popular notion in L2 acquisition; [5]), or does transfer happen over time and only for individual grammatical properties [9, 10]? These two options entail different predictions from the outset. In the first scenario, learners start with a complete grammar (a *fully specified* one), even if it is not yet targetlike. In the second, the L3 grammar starts out being largely underspecified (i.e., ambiguous with respect to most grammatical constructions), and becomes more specified over time.

In this study, we address the factors that condition transfer source selection (*a* above), but the main novelty rests on how our methodology attempts to weigh in on the full vs. property-by-property transfer debate (*b* above). We examine English as an L3 of Spanish/Catalan bilinguals with controlled, counterbalanced order of acquisition and a wide range of bilingual dominance, at the very beginning of L3 development in a constrained setting of L3 exposure (see Method section below). To the best of our knowledge, this is the first study to offer results across three languages, for the same speakers, from a wide array of unrelated, novel domains of morphosyntax, four in total (Differential Object Marking, Determiner + Proper nouns, VSO word order, causative structures). These four properties come together with data from two other domains of grammar reported on in other work, collected at the same time in the same population: definiteness effects [11] and negative polarity [9, 12]. As a result, we rely on evidence from six domains of grammar in the same learners, tested after eight weeks of a purposefully designed English language course, to adjudicate between claims of full versus property-by-property transfer in L3/L*n* morphosyntax. This is an improvement from the vast majority of previous studies, which have looked at single domains of grammar [12, 13 for review] and are thus unable to estimate the extent of transfer (full vs. partial) with any degree of confidence.

An important methodological issue is how to identify transfer, understood as the copying of linguistic representations from one language to another. Because copying a grammatical representation from language X into language Y will most likely result in behavior in Y that mirrors X, this will be hard to distinguish from a momentary intrusion of language X while processing language Y (without the copying taking place). One arguably reliable criterion might then be systematicity: if behavior in Y consistently mirrors X, then it is more likely that this influence has taken place at the level of linguistic representation. However, acquired linguistic knowledge *of any kind*, transferred or not, yields systematic behavior. Simultaneously, any non-native grammar gets closer to its target as time goes by, which means that it becomes harder to distinguish linguistic knowledge that has been transferred from that which is developed from normal input, without the involvement of previous languages [14]. Two conditions attenuate this confound. The first is looking for errors, which can be more easily checked against (and attributed to) previously acquired languages. The second is testing learners as close as possible to the first time they are meaningfully exposed to the L3/L*n*, when they simply cannot have received the necessary input to have developed novel grammatical representations.

Another good reason to test as close to first exposure as possible (in what have been called the *initial stages* of L3 acquisition) is that this is the period of L3/L*n* development where all major theories overlap in scope. Because all models (can) make direct claims about this period, it is preferable to collect data at this point than at later stages of development if our aim is to test these theories against one another.

## 2. Full vs. property-by-property transfer in L3/L*n* acquisition

Available models for L3 transfer selection can be distinguished by their claims along two axes: the source and the timing/extent of transfer. Some proposals—which we will call default models—argue for a default primacy effect of either the L1 [e.g., 15] or the L2 [e.g., the L2 Status Factor, L2SF; 16, 17]. Other proposals—non-default models—claim that both the L1 and L2 have, in principle, the same potential for transfer. With respect to the timing and extent of transfer (i.e., when and what amount of the source grammar is copied over), default models are effectively ambiguous. In principle, L1 or L2 default transfer can mean that the whole grammar is copied over as the first version of the L3 grammar, or that transfer takes place property-by-property, over development, but it always comes from the same language.

Further distinctions can be made within non-default models, on two fronts. The first is the variables they attribute transfer selection to. The second is the extent and timing of transfer itself, that is, whether or not it is wholistic early on (full transfer models) or property-by-property over the course of development. An example of the latter is the Cumulative Enhancement Model [CEM; 18], which assumes property-by-property transfer at any time in development, where transfer is warranted under two conditions: (i) the learner is at a point in L3 development where specification for a particular grammatical property is needed, and (ii) the L1 and/or L2 specification for that property is facilitative (i.e., it matches the target L3 property). Therefore, the CEM precludes the possibility of influence that would result in an “incorrect” (i.e., non-targetlike) specification.

The Typological Primacy Model [TPM; 8, 19, 20] exemplifies the other type of non-default proposal: a full transfer L3 model. It argues that transfer selection is motivated by how similar, overall, the underlying grammatical structure of the L3 is to the grammar of each previous language. Under the TPM, the full grammar of one of the previous languages is transferred as soon as this (implicit) comparison is completed. Once transferred, this copy of the L1 or the L2 constitutes the learner’s initial hypothesis (or initial specification) of the L3/L*n* grammar, which will be reconfigured throughout the course of acquisition to better deal with the L3 input. The model includes specific claims about how this evaluation takes place. After initial exposure, the L3 input is systematically compared to the relevant representations of the L1 and the L2, following a particular order of linguistic domains [a hierarchy; 8, 20]. See below for details).

The Linguistic Proximity Model [LPM; 10, 21] sits somewhere between the CEM and TPM. With the CEM, it maintains that transfer happens property-by-property over time. However, it argues that a transfer source is selected based on L3 to L1/L2 comparisons for each linguistic property, irrespective of whether either of them provides a perfect match. Unlike the TPM, the LPM does not expect the first L3 grammar to be fully specified.

## 3. The present study

Our study attempts to shed significant light on current debates over L3 transfer selectivity and extent, by addressing the following research questions:

1. Is there evidence of full transfer when we bring data from multiple domains of grammar together?
2. Which model of L3 transfer is in the best position to explain the results?

The TPM predicts an initial L3 grammar that is fully specified (because it has been copied from a previous grammar). Therefore, the model would not be supported if, for some or all of the grammatical domains tested, we do not find evidence of transfer from a single source language. However, even if we did find that all properties seem transferred from the same language (full transfer), this would still not constitute definitive support for the TPM. The reason is that this model makes specific predictions about *which* previous language (L1 or L2) will be selected for any given L1-L2-L3 combination. It is thus important to establish beforehand what language is predicted to transfer into L3 English in our study. This can be done applying the TPM’s cue hierarchy, a component that stipulates the order in which the different aspects of these languages will be compared: first the lexicon, then phonology and/or phonotactics, then functional morphology and, finally, syntax proper (basics, e.g., default word order). Importantly, the model establishes that similarity at a given level is not considered until the parser moves on from the previous one. The following section discusses an application of the hierarchy to our language combination (Catalan-Spanish-English), as well as the predictions of other models, where available.

### 3.1 Predictions

As argued in previous work with the population in this study (Spanish/Catalan bilinguals with English as an L3), lexical similarity is likely to favor neither of the two previous languages, as the vast majority of lexical overlap will come from the presence of words of Romance (from Middle French) and Greek origin in English, which will have cognates in both Catalan and Spanish. However, the next level, phonology, is argued to offer sufficient information to adjudicate between the two, as Catalan exhibits more proximity to English in this domain than does Spanish [e.g., 9, 11, 22, 23]. First, at the segmental level Spanish has a very reduced set of word-final legitimate consonants (/r/, /s/, /d/, /l/, /θ/, /n/); Catalan, in contrast, is closer to English in also admitting in this context palatal consonants, bilabial consonants or dental consonants. Second, as noted by Prieto et al. [24], both English and Catalan, but not Spanish, have vowel-reduction processes in unstressed positions. Both of these properties shared by English and Catalan are typical of stress-timed languages. Indeed, some researchers [e.g., 25] have classified Catalan as an intermediate language, exhibiting partial properties of stress-timed languages, represented in its pure form by English, while Spanish would squarely fit with syllable-timed languages.

Segmental-level criteria also favor Catalan over Spanish. While still far from the 13 vowels of some standard varieties of English, Catalan has a larger vowel inventory than Spanish (7 vs. 5 vowels), including a larger set of central vowels where the unstressed schwa is shared with English [26]. The spectral distinctions (F1 and F2 values) for high- and mid-front vowels are also similar between English and Catalan. While there is very little research directly comparing English, Spanish and Catalan phonologically outside of rhythm, a recent study [27] has shown how the similarity in the vowel system between English and Catalan might help L1 English learners of Catalan acquire the target vowel system. Still on the segmental front, Catalan and English share several consonantal sounds and phonological contrasts that are not present in Peninsular Spanish, including /b/ versus /v/, /s/ versus /z/, /ʃ/, or /dʒ/. The TPM would predict that these characteristics of the incoming L3 English input should result in a more frequent activation of mental representations associated with Catalan (as compared to those of Spanish), yielding a comparatively higher activation for this language. This ultimately leads to its selection as the transfer source, to form the basis of the first L3 English grammar of these learners.

Evidence of different sources of transfer in different grammatical domains would constitute evidence against the stipulation of full transfer defended by the TPM. Evidence of Spanish-like representations across the board, while supporting full transfer, would defy, and call for a revision of, the model’s hierarchy. As for other theories, default L1/L2 models can be ruled in or out given the counterbalancing of our participant groups in terms of L1/L2 order (and relative dominance): if order of acquisition does not modulate performance, this would be difficult for a default model to explain. Since they expect transfer selection to be evaluated for each property individually, the CEM and LPM would have difficulty explaining systematic evidence of transfer from only one of the languages (Spanish or Catalan), especially since this would sometimes be facilitative and sometimes not—a situation not contemplated by the CEM. Beyond extent of transfer, we would need specific predictions from these models to evaluate them, about which language transfers for each grammatical property. While we are able to derive these predictions straightforwardly for the CEM (the source should be whatever matches the target L3 English grammar for that property, or transfer will not obtain in the first place), we do not at present have the mechanisms to do this for the LPM, which means that the model itself cannot be tested beyond its stipulation of property-by-property transfer, equally shared by the CEM.

## 4. Linguistic properties

We probed four independent grammatical properties that differ between Catalan and Spanish, either in their expression/instantiation or in their presence in the grammar altogether: (1) differential object marking with noun phrases (DOM), a property of Spanish not shared by Catalan in the context we examine (or by English at all); (2) determiners (articles) preceding proper nouns, present in Catalan but not in Spanish (or English); (3) the word order (V)erb-(S)ubject-(O)bject, which Spanish allows but Catalan and English disallow; and (4) constructions of the type causative verb-(D)eterminer (P)hrase-infinitive, which is grammatical in Spanish (and English), but not in Catalan (*Note*: PFTV = perfective [auxiliary] verb; DAT = dative marker; DOM = differential object marker; D = determiner; N = noun; DP = determiner phrase; NP = noun phrase):

(1) a. Juan ha leído *(a) este autor.          *Spanish*

Juan has read DOM this author

b. John has read (*to) this author.          *English*

c. En Joan ha llegit (*a) aquest autor.          *Catalan*

(2) a. (*El) Juan está aquí.          *Spanish*

the Juan is here

b. (*The) John is here.          *English*

c. *(En) Joan és aquí.          *Catalan*

(3) a. Ha leído Juan un libro.          *Spanish*

has read Juan a book

b. *Has read John a book.          *English*

c. *Ha llegit en Joan un llibre.          *Catalan*

(4) a. Juan hizo a María salir.          *Spanish*

Juan made DOM María exit

‘Juan made María go out’

b. John made María get out.          *English*

c. *En Joan va fer (a) la Maria sortir.          *Catalan*

the Joan PFTV make (DAT) the Maria exit

In Catalan, the presence of a definite article is obligatory when the proper noun (name) appears in argument position. Whether names combine with articles or not is standardly analyzed as a syntactic property that specifies whether the D head of a constituent containing a proper noun is filled by the noun itself through movement (5a), or has to be filled by an independent element (5b). The underlying assumption in this account is that any noun must be licensed by a determiner in order to act as an argument [28–31, among others], with languages differing with respect to whether the D head is filled by N-movement or not.

(5) a. [DP     N+D [NP]]          *Spanish and English*

b. [DP     D_[art]_ [NP N]]               *Catalan*

The second property we examine is the position of a causee DP within causative structures. In Catalan, this causee must appear after the infinitive, and gets dative case [32–35], as in (6):

(6) Vaig fer (*a la Maria) comprar un cotxe **a la Maria**.

PFTV._1SG_ make DAT the Maria buy._INF_ a car DAT the Maria

‘I made Maria buy a car’

The standard explanation of this restriction on word order [36] is related to case assignment. In this analysis, the causee cannot get case-assigned by the causative verb *fer* ‘make’, so it must stay within the infinitival clause, where it gets assigned an oblique case.

In the case of Spanish DOM, there is consensus that, for NPs, the animacy and the specificity of the argument are relevant to determine whether marking is assigned or not [37–41], as well as the degree of affectedness of the object with respect to the predicate [42–44]. DOM is only compulsory in specific animate nominals (7a), while nonspecific readings tend to carry no marking (7b; see [45] for partial exceptions):

(7) a. Busco a una secretaria.

search._1SG_ DOM a secretary

‘I am looking for a particular secretary’

b. Busco una secretaria.

search._1SG_ a secretary

‘I am looking for anyone who can work as a secretary’

Finally, the word order VSO [46–48] depends on a combination of syntactic and pragmatic factors. Syntactically, Ordóñez [49] proposes that Spanish contrasts with Catalan in that it can license subjects in a low functional position above the V(erb) P(hrase):

(8) [Auxiliary [SubjectP     [VP]]]          *Spanish*

However, licensing in that position is not the only condition for VSO. Leonetti [50] shows that the structure is associated to a specific information structure: a thetic structure with wide focus—that is, one in which the whole clause is in focus without a topic/comment partition, and no element gets narrow focus. Thus, a sentence like (9) in Spanish should not be grammatical if any of the elements of the clause are given any type of emphasis over the rest.

(9) Ha ganado España el mundial.

has won Spain the world-cup

‘Spain has won the World Cup’

These four properties provide a good testing case for two reasons. The first is they are autonomous, that is, independent of each other. The second is that they present a wide range of relations with semantics, pragmatics and/or phonology. Taken together, these characteristics place our examined properties in a stronger position when it comes to generalizing claims to the whole of the grammar. Table 1 presents a summary of the properties, with an indication of their acceptability/grammaticality in each language. Note that we list grammaticality/acceptability in English here only for reference, as participants had very little to no actual L3 English input (depending on the property) on these constructions.

**Table 1:** Summary of the properties, with acceptability/grammaticality in each language according to theoretical descriptions of standard varieties (note: blue = acceptable/grammatical; brown = unacceptable/ungrammatical).

Property	Spanish	Catalan	English
DOM			
VSO			
Det+Name			
Causative + DP			

An anonymous reviewer points out that it might be problematic to test syntax-semantics or syntax-pragmatics interface properties along purely syntactic ones, because violations of the former are more subtle, and the presence of more formal violations could lower the participants’ threshold sensitivity to the subtleties implied in evaluating whether a sentence that contains interface properties is felicitous. It might indeed be the case that part of the complexity in our data, which we discuss at length in section 7, stems from adjustments and readjustments in sensitivity to different types of violations as a result of the mixture of conditions requiring purely syntactic evaluations vs. pragmatic/semantic licensing. Whether this is the case remains an empirical question, and a methodological word of caution for future research.

## 5. Method

This study was carried out in accordance with the recommendations of the Research Ethics Committee at the University of Reading. The protocol was approved by the School of Psychology and Clinical Language Science’s Research Ethics Committee, and all subjects gave written informed consent in accordance with the Declaration of Helsinki.

### 5.1. Learning context and L3 input

In order to focus on an early stage of L3 development, we designed a two-month course for true *ab initio* learners of English [see 9, 12]. Designing and administering this course allowed us to (a) narrow down our population sampling to people without previous experience with naturalistic exposure or formal instruction in English; and (b) ensure that they learned the minimum vocabulary required to undertake all experimental tasks administered at the end of the course, while receiving virtually no exposure to the critical grammatical properties. Although English is a common second language across generations in many European countries, this is not true of Spain (and, by extension, of Northeast Catalonia, where our study was conducted), where the older generations did not have access to formal instruction in English during their school years—French being more popular at the time. For this reason, we targeted individuals around the age of 50, who were unlikely to have had formal education in English as a foreign language.

### 5.2. Participants

We tested a group of 40 Catalan-Spanish bilinguals who were *ab initio* learners of L3 English. Participants had to meet the following inclusion criteria:

Be speakers of Catalan and Spanish born and raised in Osona, Catalonia (see [51] for the specific sociolinguistic information of this area).
Be *ab initio* learners of English (i.e., no previous exposure, formal or informal, to English).
Complete the task in the three languages (Catalan and Spanish tested after English, counterbalanced in order across participants).

Overall, out of the 73 participants who agreed to attend the testing sessions at the end of the two-month course, 33 (45.21%) had to be excluded for not meeting one (or more) of the criteria above. A large proportion of these (*n* = 19) were excluded because they were not *ab initio* (low exposure) learners (see (9), for discussion), but rather low proficiency learners with 12–48 months of previous instruction in English. Table 2 summarizes the information of the 40 remaining participants.

**Table 2:** Participant information.

**L1**	Catalan (*n* = 22) or Spanish (*n* = 18)
**Age**	50.8 (5.5)
**Sex**	Female (*n* = 28) or Male (*n* = 12)
**L3 Proficiency**	5.3 (1.8) out of 60
**LD**	41.32 (*SD* = 105.54; range: -203 to 217)

Standard deviations and ranges, where applicable, between parentheses. (*Note*: LD = language dominance; L3 Proficiency = English proficiency [Oxford Quick Placement Test]. See subsection below for details).

#### 5.2.1. Language dominance

Since our participants were all early (child) sequential bilinguals of Catalan and Spanish (L1 Spanish-L2 Catalan, *N* = 18; L1 Catalan-L2 Spanish, *N* = 22), their relative dominance between both languages as measured through their amount and context of use varied as a function of complex circumstances in their life history, and was not always directly predictable from their L1—although, as expected, it was on average (*F*(1, 38) = 8.60, *p* < .01). For this reason, and since the context of acquisition of early bilinguals effectively neutralizes the main factors argued to underlie proposals of default transfer from the L1 or the L2 (e.g., [52]; see [20] for discussion), we decided to use dominance instead of order of acquisition in our analyses as the finer-grained measure out of two partially correlated factors, to avoid issues of collinearity of predictors.

To ensure that our analyses did not miss potential (distinct) effects of order of acquisition or language of instruction [e.g., 53], we ran a first model within each Condition data set in which we included L1 and Language of instruction, as well as Subcondition and Dominance (and all interactions between these four variables) as fixed factors, with random intercepts for Subjects and Items. No analysis revealed significant main effects or interactions involving L1 (max. *z* = -1.52; min. *z* = 0.03) or Language of Instruction (max. *z* = 1.80; min. *z* = 0.03) with Subcondition. One of the models (Det + Name in the Spanish data) did not converge when including either of these factors. Another model (Word order in the Spanish data) revealed a significant interaction between L1 and Language of instruction, suggesting that ratings were lower overall when Spanish was the L1 and this was also the language of instruction. However, post-hoc comparisons did not confirm this result (i.e., the interaction was non-significant). In the model for Word order in the English data, L1 was significant, suggesting that ratings were higher overall when the L1 was Spanish. One other model (DOM in the Catalan data) contained complex three- and four-way interactions between these two factors, Dominance, and Subcondition, suggesting that Spanish L1 or instruction in this language compensated for a tendency to assign lower ratings to sentences with DOM the more Catalan-dominant a participant was. Finally, the model for DOM in the Spanish data yielded a significant effect of L1 and the interaction of Dominance with Language of instruction. When the L1 was Spanish, the ratings were lower, and when the instruction happened in Spanish, more Catalan-dominant participants gave higher ratings. Including random structure in these models of the type that we have used in the final analyses (with random slopes for each fixed factor and interaction, see below) would raise the number of random-effects parameters above the number of observations, which would confound the residual variance with variance explained by the model’s random structure—meaning that models could not be reliably computed. To ensure that the use of appropriately complex random structures provided the right sensitivity to capture potential effects of Dominance, we decided to leave out L1 and Language of Instruction after these exploratory analyses.

Some studies focusing on (adult) L3 acquisition by simultaneous and early child bilinguals have proposed language dominance—operationalized either in terms of relative language use or relative language proficiency, depending on the study—as a deterministic factor in L3 transfer. However, the relative dearth of studies examining this variable, which have found mixed results, make the role of dominance inconclusive so far [e.g., 53–56]. To include language dominance as a continuous variable, we used the Bilingualism Language Profile [57], which measures domains and relative amounts of use between the two languages through a detailed collection of self-report questions.

The BLP generates a score within a scale between -218 and +218 calculated on the basis of aspects such as age of onset, use (proportion as well as context of use), preference and self-rated proficiency, the four main areas of the proficiency index employed by Lloyd-Smith et al. [58]. That study, which provides one of the most detailed operationalizations of dominance in the L3/L*n* literature, also made use of a Yes/No vocabulary task that complemented the self-report measures with objective proficiency data. While we are missing a more explicit focus on proficiency, we believe that self-report measures are sufficient for our current purposes of control, especially in light of the frequent (partial) correlations between objective proficiency measures and fine-grained assessments based on language exposure [e.g., 58–60]. In our coding system, -218 would correspond to absolute Spanish dominance, whereas +218 represents total dominance in Catalan. The average score for our participants was 41.32 (*SD* = 105.54; range: -203 to 217). The variable was normally distributed in our sample (*W* = 0.97284, *p* = 0.44).

Participants also took the *Oxford Quick Placement Test* [61] as a general measure of proficiency in English after the two-month course. As expected, the scores distributed towards the very lower end of the spectrum (5.3 out of 60 possible points on average), demonstrating that these participants’ overall proficiency remained low.

### 5.3. Materials and procedure

Data were collected through an Acceptability Judgement Task (AJT) conducted separately in each language (English, Catalan and Spanish). The L3 was always tested first to avoid any potential priming, and Catalan and Spanish were tested afterwards in a pseudo-randomized, counterbalanced way. A week passed between each language being tested. The AJT included 4 different conditions, which were themselves subdivided into two or more subconditions. Table 3 provides sample sentences from the English AJT. The full list of materials for all languages can be found in S1 File.

**Table 3:** Example items in the English Acceptability Judgement Task, coded by Condition and Subcondition.

**Condition**	**Subcondition**	**Example item**	**N**
**DOM**	No DOM	My mother invited my friend.	5
	DOM	*The dog attacked to this boy	5
**Det + Name**	Name	The doctor is dancing with Peter	5
	Det + Name	*The doctor is talking to the Mary	5
**Word Order**	VOS	* Draws a picture the girl.	5
	VSO	*Ate the girl pizza	5
**Causatives**	C + Pro	The girl made him buy an apple.	5
	Per + Pro	The teacher is making him write a book.	5
	C + DP	The boy made the girl read a book	5

(*Note*: C+Pro = causative verb + (clitic) pronoun + infinitive; Per+Pro = periphrastic causative verb + (clitic) pronoun + infinitive; C+DP = causative verb + full DP).

The test contained 45 critical sentences, presented in random order, distributed across four conditions and nine subconditions with five items each. To achieve an equal number of grammatical and ungrammatical items, and since these vary asymmetrically across our languages depending on the grammatical property at hand, we introduced subconditions that would provide the right balance of grammatical to ungrammatical sentences overall across our tasks. In this spirit, we compared sentences with DOM or a determiner preceding a name to (grammatical or ungrammatical) counterparts lacking these features. Sentences in the VSO condition (*Ate John an apple, only grammatical in Spanish) were compared to VOS (*Ate an apple John), with similar information-structure constraints and grammatical both in Catalan and Spanish. Finally, the causative sentences with a full DP causee were compared to two causative constructions involving (clitic) pronouns: one in which a pronoun substituted the DP in a position intervening between the causative verb and the infinitive (*John made her[pron*.*] cry*, unacceptable in both Catalan and Spanish), and one in which the causative verb was periphrastic (*They are making her[pron*.*] cry*, grammatical in both previous languages).

To complete the balance of grammatical to ungrammatical sentences, we added a variable number of distractors/fillers to each task, as necessary (English: five, all grammatical; Catalan: 15, five grammatical; Spanish: 25: five grammatical). Ungrammaticality in distractor items was mostly introduced through errors of agreement or semantic anomalies.

Participants were asked to rate the acceptability of each token on a 1 to 4 scale (1 = totally unacceptable; 4 = totally acceptable). In addition, they were given a “not sure” option. The range of 1 to 4 with an external *NA* option was selected in order to maximize informativeness of responses within the 1 to 4 range, so that middle values (e.g., 3 in a 1 to 4 scale) did not attract responses that are due to hesitation rather than genuine “gray-area” acceptability. Each item was presented individually in the middle of the screen. The AJT was conducted within the framework of a larger study [12], being one of four tasks participants took in each experimental session.

An anonymous reviewer brings up two methodological comments. First, one might argue that removing the middle point of the scale and adding a “not sure” option tackle the same potential confound and are thus redundant. In retrospect, we agree an even-numbered (5-point or 7-point) scale *in combination with* an external “not sure” option might have allowed us to capture true ambivalence/marginal acceptance with a finer degree of granularity, but we think that the overall patterns of the data would likely remain similar. The second issue has to do with not having included a correction task, which could leave us in the dark about whether rejections of ungrammatical/unacceptable sentences are done for the right reasons. While we agree in general terms, we see two problems with that approach. First, by accepting only correct rejections of ungrammatical sentences if they are followed by an accurate correction, one is enacting an implicit assumption that “yes” responses to grammatical items are also guided by the right grammatical parse (and not by chance, a mis-parse on the basis of a structure in their interlanguage grammar other than the target, etc.). Ultimately, explicit behavioral responses are the outcome of a process that depends on numerous nuisance variables beyond the underlying knowledge base that is consulted/operated on. The second problem is that certain types of violations, especially those that do not involve strictly formal elements (or require interface assessment) are sometimes hard to pinpoint and thus provide corrections for, even by native speakers. We thus think that validating rejections through correction may be too conservative, as it runs the risk of underestimating the grammatical knowledge of the speaker.

### 5.4. Data analysis

Data were analyzed using ordinal mixed regression models with crossed random effects for subject and items [62], using the packages *ordinal* [63] to fit the models and *emmeans* [64] for post hoc comparisons of treatment effects, in the statistical software *R* [65]. For each subset, we fit a model with main effects of Subcondition and Dominance as well as their interaction as fixed factors. For the random structure, we opted for a parsimonious model employing backward selection. We started with a maximal model, including random intercepts for subjects and items, as well as random slopes for all fixed factors within each of the random factors. We then started to reduce the structure’s complexity by removing a factor at each step and comparing the old and new model(s), until there was a significant loss in the goodness of fit as determined by a likelihood ratio test (the significance level was set at a conservative 0.2) [66].

While our main analyses focus on condition effects within each language, we also report interaction analyses, where the data sets for the same condition in English and Spanish, on the one hand, and English and Catalan, on the other hand, were combined to run models that included Language (of testing) and its interaction with Subcondition as fixed factors. This was suggested by the editor and an anonymous reviewer. Although we agree that a significant effect of Subcondition *without* a significant Language by Subcondition interaction in these data sets (i.e., equal size effects) would be more directly suggestive of transfer, we worry that framing this as a necessary rather than a sufficient criterion might be somewhat reductionist, because it implicitly assumes that acceptability judgements reflect grammatical competence directly without intervening noise from other variables, both representational (e.g., lexical knowledge is not fully reliable in beginner learners) and performance-related. As the reviewer her-/himself notes, there might be transfer in the presence of an interaction in the data, simply because the effect happens to be smaller in, e.g., English than it is in Catalan, even if they are qualitatively similar. For these reasons, we report and interpret these analyses here for completeness, although we note from the outset that we believe they do not provide the best insight into the theoretical question at hand.

Formulas for the final, converging models in each dataset are reported in S2 File, together with full model outputs and, where applicable, output tables for post hoc comparisons. All data and analysis scripts, including interaction analyses, are available at the first author’s OSF repository (https://osf.io/64eb3/).

## 6. Results

### 6.1. Determiner + Name

**Fig 1:**
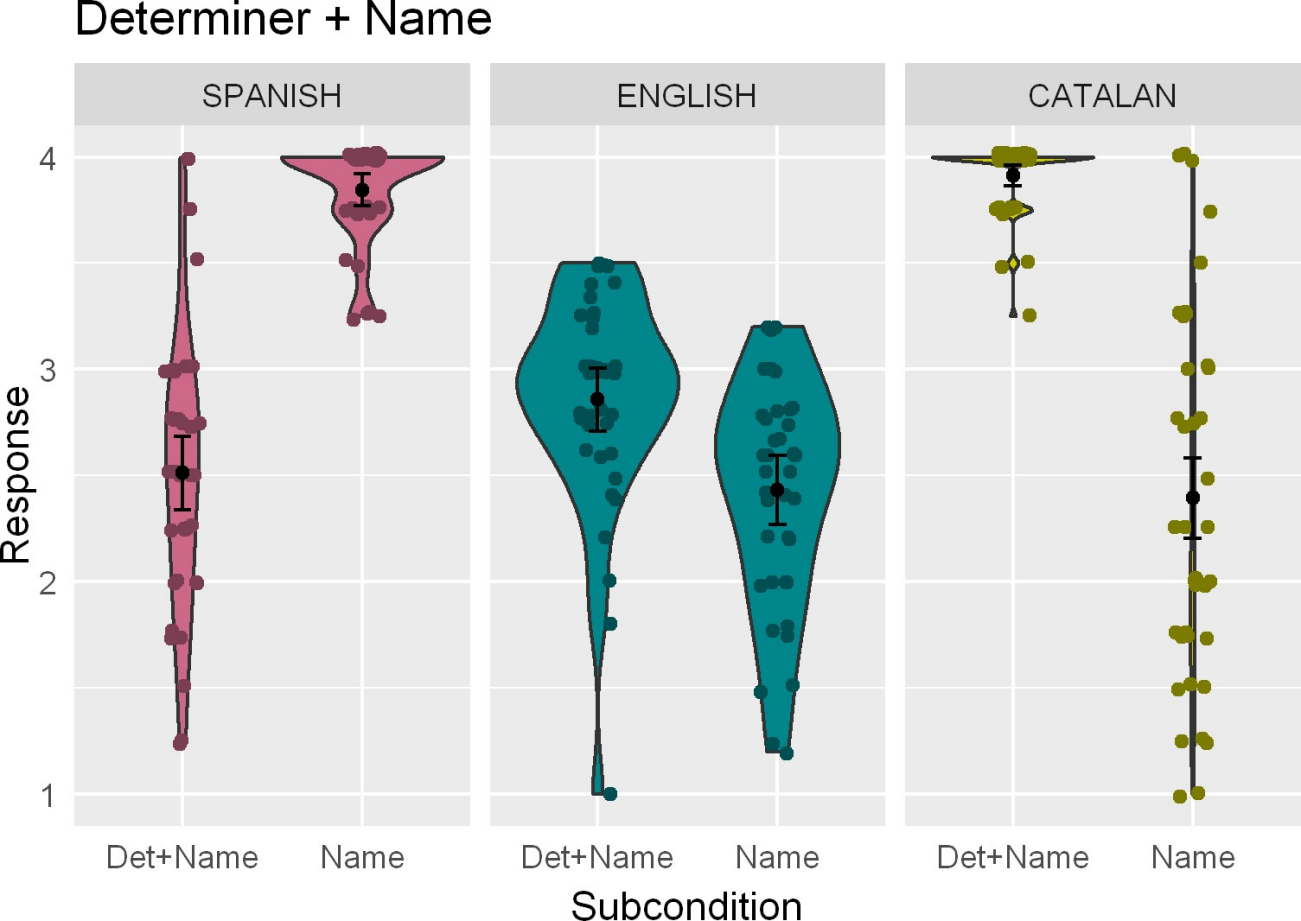
Kernel density (violin-) plots indicating the mean rating and the 95% confidence interval of the population mean for the Determiner + Name condition across the three languages. Dots indicate individual means.

Average ratings to sentences in the Determiner + Name condition across the three languages can be seen in Fig 1. In the Catalan data, sentences without a determiner preceding a proper noun in argument position received significantly lower ratings than their counterparts without a determiner (*β* = -4.36; *z* = -9.44; *p* < .001). There were no effects of Dominance, neither as a main effect (*β* = -0.53; *z* = -0.31; *p* = .76) nor in its interaction with the Subcondition factor (*β* = -0.30; *z* = 0.18; *p* = .86). In Spanish, this preference was reversed: sentences without a determiner preceding a name received significantly higher ratings than sentences with it (*β* = 3.41; *z* = 9.40; *p* < .001). Also, Dominance played a role here (*β* = 1.69; *z* = 2.30; *p* < .001): overall ratings were significantly higher in more Catalan-dominant bilinguals. The interaction between Dominance and Subcondition was not significant (*β* = -0.98; z = -0.80; *p* = .43). Finally, English sentences without a determiner + name construction received significantly lower ratings than those with it (*β* = -0.72; *z* = -0.19; *p* < .001), as in Catalan and different from Spanish. Neither Dominance (*β* = -0.62; *z* = -1.11; *p* = .27) nor its interaction with Subcondition (*β* = -0.28; *z* = -0.35; *p* = .73) yielded significant estimates.

#### 6.1.1. Interaction analyses

The interaction analysis for the Catalan-English joint data set revealed main effects of Subcondition (*β* = -0.71; *z* = -3.69; *p* < .001) and Language (*β* = 3.67; *z* = 8.64; *p* < .001), with lower ratings to bare-name sentences overall and higher ratings in Catalan as compared to English. These main effects were qualified by a Language by Subcondition interaction (*β* = -3.73; *z* = -3.69; *p* < .001) indexing a difference in the size of the effect, larger in Catalan (as Tukey post-hoc analyses find a significant Subcondition effect in both languages; English: *β* = 0.71; *z* = 3.69; *p* < .01; Catalan: *β* = 4.44; *z* = 11.48; *p* < .001).

In the Spanish-English joint analysis, the model also contains significant main effects of Subcondition (*β* = -0.72; *z* = -3.71; *p* < .001) and Language (*β* = -0.57; *z* = -2.87; *p* < .01), with lower ratings to bare-name sentences overall and lower ratings in Spanish as compared to English. These main effects were qualified by a Language by Subcondition interaction (*β* = 4.02; *z* = 11.13; *p* < .001) indicating that the (significant) effect of Subcondition takes opposite directions in each language (Tukey post-hoc analyses; English: *β* = 0.71; *z* = 3.69; *p* < .01; Spanish: *β* = -3.31; *z* = -11.04; *p* < .001).

### 6.2. Word order

**Fig 2:**
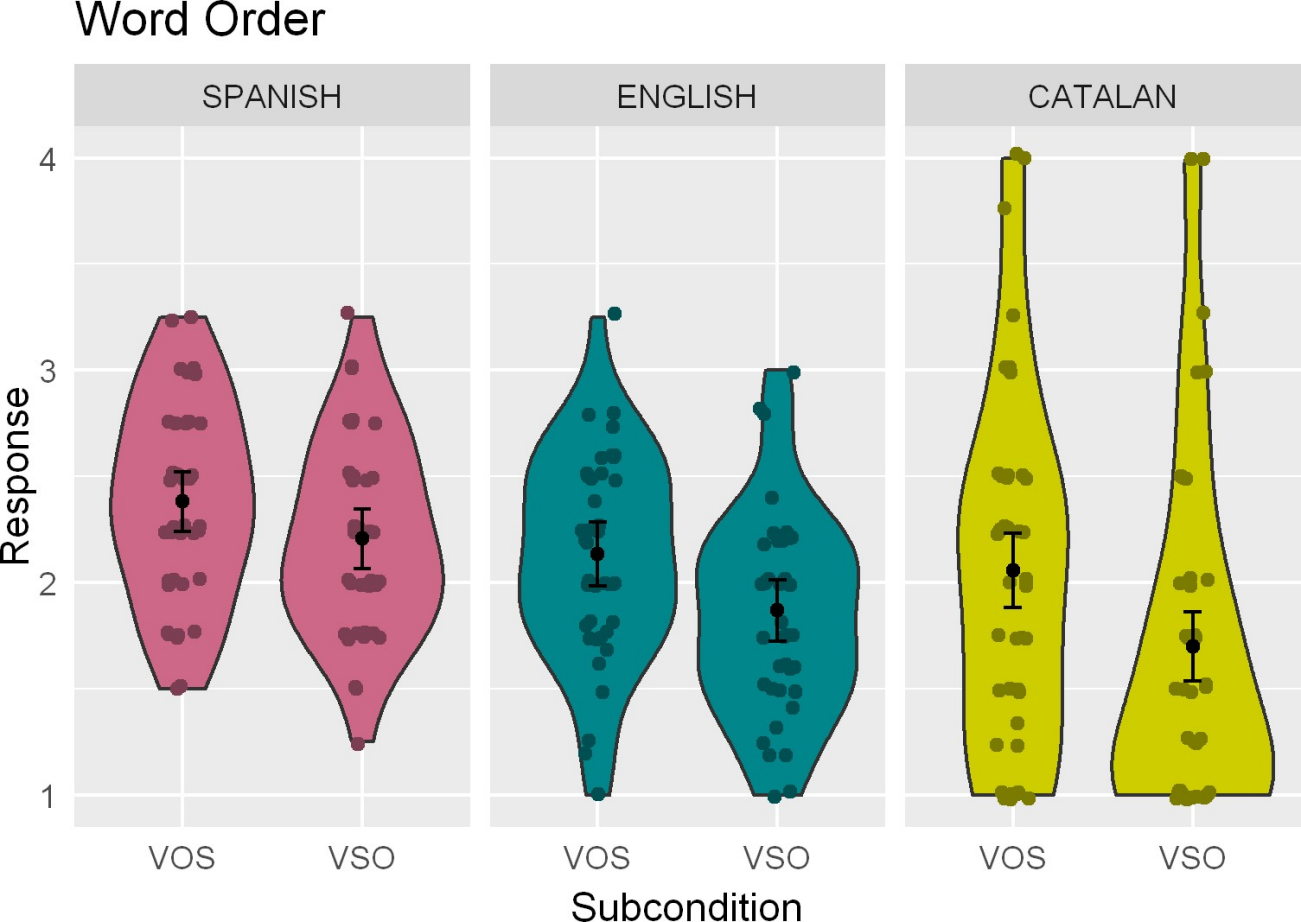
Kernel density (violin-) plots indicating the mean rating and the 95% confidence interval of the population mean for the Word order condition across the three languages. Dots indicate individual means.

Fig 2 plots average ratings to sentences in the Word order condition across the three languages tested. In Catalan, participants provided significantly lower ratings in sentences with a VSO structure as compared to those with VOS (*β* = -1.22; *z* = -3.58; *p* < .001), although both subconditions received low ratings overall—the same being true of the Spanish and English data. Dominance did not play a role in Catalan (main effect: *β* = 0.35; *z* = 0.22; *p* = .82; interaction with Subcondition: *β* = -0.44; *z* = -0.21; *p* = .84). In the Spanish data, both VOS and VSO sentences received comparable ratings (*β* = -0.33; *z* = -1.41; *p* = .16). This was true irrespective of Dominance (interaction: *β* = -0.71; *z* = -0.79; *p* = .43), which did not affect ratings overall either (main effect: *β* = -0.01; *z* = -0.01; *p* = .99). Responses to the English sentences followed along the lines of those for the other two languages, with low ratings overall that were nonetheless significantly lower for VSO sentences (*β* = -0.59; *z* = -2.93; *p* < .01), as in Catalan and different from Spanish. The main effect of Dominance (*β* = 0.04; *z* = 0.07; *p* = .94) was not a significant predictor, although its interaction with Subcondition (*β* = 1.62; *z* = 1.84; *p* = .07) was marginally significant. This reflects the fact that ratings to VSO sentences were slightly higher by those participants with higher Catalan dominance.

#### 6.2.1. Interaction analyses

The interaction analysis for the Catalan-English joint data set contained a main effect of Subcondition (*β* = -0.59; *z* = -2.92; *p* < .01), reflecting lower ratings for VSO sentences in both languages. The Language by Subcondition interaction was not significant (*β* = -0.41; *z* = -1.28; *p* = .20), suggesting that the effect is both of equal sign and comparable magnitude in English and Catalan. Tukey post-hoc tests confirmed a significant effect of Subcondition in both languages (English: *β* = 0.59; *z* = 2.90; *p* < .05; Catalan: *β* = 1; *z* = 4; *p* < .001).

In the Spanish-English joint analysis, the model contains significant main effects of both Subcondition (*β* = -0.64; *z* = -3.12; *p* < .01) and Language (*β* = 0.49; *z* = 2.45; *p* < .05), indicating lower ratings to VSO sentences overall and higher ratings in Spanish, respectively. The effect of the Language by Subcondition interaction was not significant (*β* = 0.33; *z* = 1.16; *p* = .25), but the adjusted post-hoc tests confirmed the Subcondition effect only for English (*β* = 0.63; *z* = 3.11; *p* < .05; Spanish: *β* = 0.31; *z* = 1.54; *p* = .41).

### 6.3. Differential object marking

**Fig 3:**
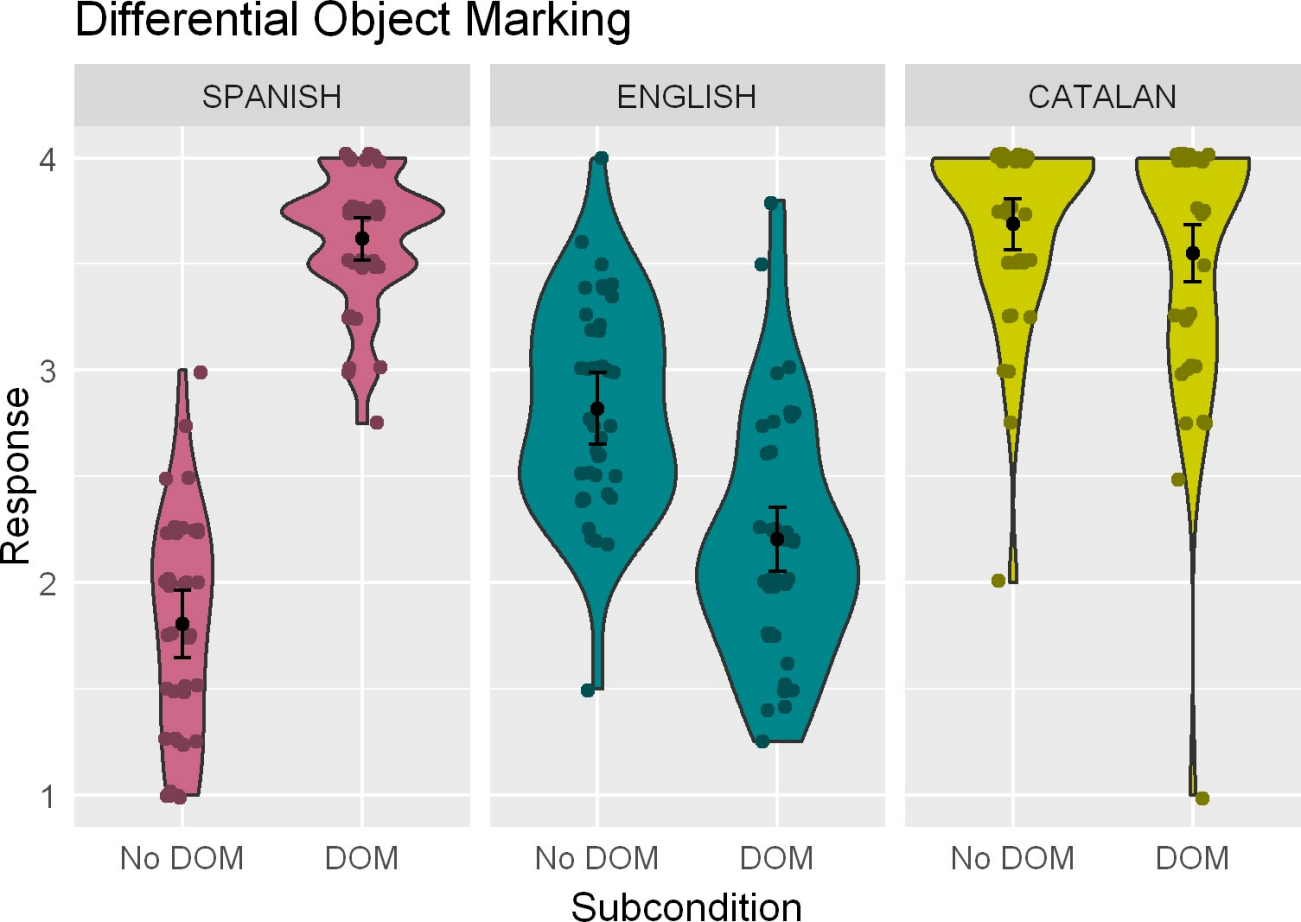
Kernel density (violin-) plots indicating the mean rating and the 95% confidence interval of the population mean for the Differential Object Marking condition across the three languages. Dots indicate individual means.

Average ratings to sentences in the Differential Object Marking can be seen in Fig 3. Participants provided comparably high ratings to Catalan sentences with and without DOM (*β* = -0.27; *z* = -0.33; *p* = .74), and they did so irrespective of Dominance (*β* = 0.34; *z* = 0.19; *p* = .85). The interaction of Subcondition and Dominance (*β* = 3.69; *z* = 1.82; *p* = .07) was marginally significant: more Catalan-dominant participants gave slightly higher ratings to sentences with DOM. Contrary to their ratings in Catalan, participants displayed a significant preference for Spanish sentences containing DOM in the relevant contexts (*β* = 3.59; *z* = 11.43; *p* < .001). This pattern was unaffected by Dominance (main effect: *β* = 0.40; *z* = 0.58; *p* = .56; interaction: *β* = -0.30; *z* = -0.30; *p* = .76). They showed the opposite preference in English, however, where sentences with DOM received significantly lower ratings (*β* = -1.09; *z* = -5.31; *p* < .001). Dominance did not play a role in explaining the data (main effect: *β* = -0.29; *z* = -0.42; *p* = .674; interaction with Subcondition: *β* = 0.68; *t* = 0.81; *p* = .42).

#### 6.3.1. Interaction analyses

The interaction analysis for the Catalan-English joint data set contained main effects of Subcondition (*β* = -1.05; *z* = -4.47; *p* < .001) and Language (*β* = 2.27; *z* = 7.56; *p* < .001), indexing lower ratings overall to sentences with DOM, and higher ratings to both types of sentences in Catalan. The Language by Subcondition interaction was marginally significant (*β* = 0.67; *z* = 1.82; *p* = .069). Tukey post-hoc tests confirmed a significant effect of Subcondition only in English (*β* = 1.06; *z* = 4.47; *p* < .001; Catalan: *β* = 0.38; *z* = 1.15; *p* = .66).

The model for the Spanish-English joint analysis includes significant main effects of Subcondition (*β* = -1.09; *z* = -5.43; *p* < .001), Language (*β* = -1.87; *z* = -8.57; *p* < .001), and their interaction (*β* = 4.54; *z* = 13.51; *p* < .001). These correspond, respectively, to lower ratings on sentences with DOM in both languages, lower ratings to sentences in Spanish, and the fact that there are significant effects of Subcondition in both languages but with opposite sign (*β* = 1.09; *z* = 5.43; *p* < .001; Spanish: *β* = -3.45; *z* = -13.79; *p* < .001).

### 6.4. Causative constructions

**Fig 4:**
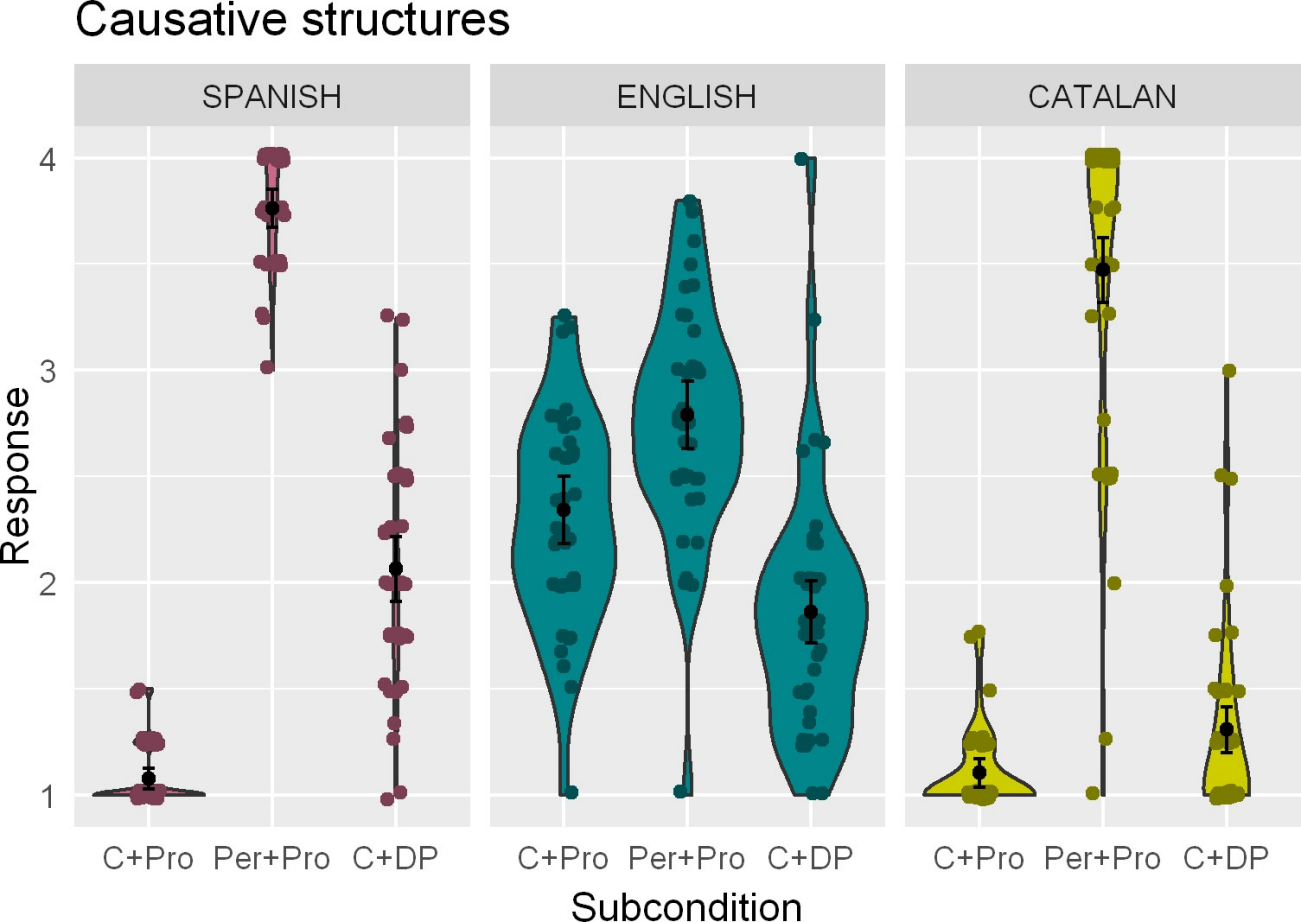
Kernel density (violin-) plots indicating the mean rating and the 95% confidence interval of the population mean for the Causative condition across the three languages. Dots indicate individual means. (*Note*: C+Pro = causative verb + (clitic) pronoun + infinitive; Per+Pro = periphrastic causative verb + (clitic) pronoun + infinitive; C+DP = causative verb + full DP).

Fig 4 shows average ratings for the different types of sentences within the Causative construction condition in each of the three languages tested. Sentences with a verbal periphrasis followed by a clitic pronoun and an infinitive (Per+Pro) received high ratings in Catalan, in stark contrast with the very low ratings of sentences where a non-periphrastic causative verb was followed by a clitic (C+Pro; difference: *β* = -7.03; *z* = -9.00; *p* < .001) or a full DP (C+DP; difference: *β* = -6.18; *z* = -7.20; *p* < .001). These two subconditions did not differ significantly from each other, although there was a numerical trend against C+Pro sentences (*β* = -0.84; *z* = -1.47; *p* = .31). Notably, while Dominance was not deterministic overall (main effect: *β* = -0.29; *z* = -0.24; *p* = .81), it did have an effect in interaction with Subcondition, so that more Catalan-dominant participants provided (even) higher ratings to Per+Pro sentences (*β* = 4.73; *z* = 2.12; *p* < .05).

Similarly, the Spanish data shows significantly higher ratings in the Per+Pro subcondition than in the other two types of sentences (Per+Pro vs. C+Pro difference: *β* = 8.30; *z* = 10.09; *p* < .001; Per+Pro vs. C+DP difference: *β* = 4.67; *z* = 6.67; *p* < .001). In this case, these two subconditions did differ from each other, with C+DP sentences receiving significantly higher ratings than C+Pro sentences (*β* = 3.63; *z* = 5.20; *p* < .001). Dominance did not play a significant role in this dataset as a main effect (*β* = 1.90; *z* = 1.02; *p* = .31). However, in the interaction with the Per+Pro subcondition (*β* = -3.88; *z* = -1.81; *p* = .07) the effect was marginally significant, showing that ratings from more Catalan-dominant participants were lower for these sentences. The interaction of Dominance and the C+DP subcondition did not yield a significant effect (*β* = -2.89, *z* = -1.49 *p* = .14).

Finally, ratings in the English data were also highest for Per+Pro sentences, which were judged more acceptable than both their C+Pro (difference: *β* = 0.79; *z* = 3.94; *p* < .001) and C+DP counterparts (difference: *β* = 1.67; *z* = 7.80; *p* < .001). These two subconditions differed from each other as well, but the pattern was the opposite to what we observed in the Spanish data: in English, it was C+Pro sentences that received significantly higher ratings when compared to the C+DP subcondition (*β* = 0.88; *z* = 4.27; *p* < .001). Neither the main effect of Dominance (*β* = -0.96; *z* = -1.32; *p* = .19) nor its interactions with the Subcondition factor (all *p*s > .81) seemed to be relevant in explaining the data.

#### 6.4.1. Interaction analyses

The interaction analysis for the Catalan-English joint data set contained significant main effects of Subcondition (C+Pro to Per+Pro: *β* = 0.82; *z* = 3.33; *p* < .001; C+Pro to C+DP: *β* = -0.90; *z* = -3.79; *p* < .001), indicating that C+Pro sentences were rated higher than Per+Pro but lower than C+DP, and Language (*β* = -3.88; *z* = -9.31; *p* < .001), indicating lower ratings in Catalan. The Language by Subcondition interaction was significant (C+Pro to Per+Pro: *β* = 5.85; *z* = 12.27; *p* < .001; C+Pro to C+DP: *β* = 2.20; *z* = 4.87; *p* < .001). Tukey post-hoc tests confirmed significant effects of Subcondition in all relevant contrasts in both languages (smallest z = -3.17, max. *p* = .019), although they differed in sign in the C+Pro to C+DP contrast, where the former was rated higher in English but lower in Catalan. The model also contained a significant triple interaction between Subcondition, Language and Dominance (*β* = 4.08; *z* = 2.36; *p* < .05), indicating higher ratings to Per+Pro sentences in Catalan by more Catalan-dominant participants.

For Spanish and English, the model for the joint data set also contained significant main effects of Subcondition (C+Pro to Per+Pro: *β* = 0.81; *z* = 4; *p* < .001; C+Pro to C+DP: *β* = -0.87; *z* = -4.28; *p* < .001), indicating that C+Pro sentences were rated higher than Per+Pro but lower than C+DP, and Language (*β* = -4; *z* = -9.97; *p* < .001), indicating lower ratings in Spanish. The Language by Subcondition interaction was also significant (C+Pro to Per+Pro: *β* = 6.52; *z* = 13.55; *p* < .001; C+Pro to C+DP: *β* = 4.36; *z* = 9.88; *p* < .001). Again, Tukey post-hoc tests confirmed significant effects of Subcondition in all relevant contrasts in both languages (smallest z = -4, max. *p* = .0003), although they differed in sign in the C+Pro to C+DP contrast, where the former was rated higher in English but lower in Spanish. There were no further main effects or interactions.

## 7. Discussion

The main objective of this study was to bring together several domains of grammar to adjudicate between full versus property-by-property models of transfer in L3 acquisition. Our results do not seem to support either set of models unambiguously. The TPM predicted that the Catalan grammar of these speakers would have been copied over as the initial hypothesis for the L3, affecting all domains. While we see some results more clearly suggesting influence from Catalan (e.g., in the Det+Name and Word order conditions), can our data as a whole be taken as consistent with full transfer? Showing a good deal of effects from Catalan can also be consistent with property-by-property transfer models, such as the LPM. Alternatively, then, do the data better support property-by-property transfer? Not necessarily. The more complex datasets, Causatives and DOM, are not clearly suggestive of a predominant influence from a single language, and a hybrid transfer pattern is not easily recoverable either Overall, failure to support one or the other type of model does not mean de facto support for the other.

In this section we engage with the complexity of the data and offer what we believe is a reasonable way to understand them. We discuss our results against the specific prediction of the TPM: the first version of these speakers’ L3 grammar should be a copy of their (L1 or L2) Catalan grammar. We consider several potential qualifications/confounds that might explain the data starting out from this assumption. We cannot do the same for property-by-property models, since we lack specific predictions about transfer selectivity (Spanish or Catalan) for each property. However, our discussion of how well the results for each property align with a hypothesized first instantiation of the property that matches the Catalan specification provides at least half of the assessment that would be needed for models such as the LPM.

Perhaps one of the most interesting yet non-central results of this study is that the data for the Catalan and Spanish of our participants did not always conform to the descriptive literature that section 4 above is based on. We will engage with these results here for the sake of exhaustiveness, but two important things should be highlighted. The first is that these deviations from expected behavior did not confound the sources: however different from description, a contrast between Catalan and Spanish is retained in all four conditions. The second is that these differences add significant value to our methodology: since we tested the same multilingual participants in all three languages, the Catalan and Spanish data sets constitute the maximally controlled baseline for the evaluation of transfer effects for each participant’s own L3 English data. Our study is ultimately about how the L1 and L2 grammars of these speakers constrained their initial L3 grammar. This means that whether their Catalan or Spanish grammars conform to theoretical description is interesting in and of itself, but largely irrelevant to our (L3) research questions. In fact, even potential CLI between their Spanish and Catalan grammars is a distracting issue, to the extent that it does not result in alignment between them, which would effectively confound the sources. What matters to test our theories of L3 transfer is *what* these speakers have available in each of their previous languages, whether it is different enough for us to be able to identify the source, and how this previous knowledge is reflected in their L3 judgements.

### 7.1. Determiners with names

Irrespective of order of acquisition and dominance, participants assigned significantly higher ratings to English sentences where names were introduced by a determiner, as compared to sentences with bare names. This pattern replicated what we found in the Catalan data, and was directly opposite to the results of this condition in Spanish. Interaction analyses point in the same direction (Catalan-English alignment), although they show that the effect is smaller in English than it is in Catalan.

As for judgements in Catalan and Spanish, we expected lower ratings for Det+Name in Spanish and bare name in Catalan (both at around 2.5 average), since these are standardly described as ungrammatical. Let us consider some probable causes for these results. In Spanish, and given that this is a contact situation, the most immediate suspect is an influence of Catalan on the Spanish native variety of these speakers. If assumed to be bidirectional, this would conversely explain why they rate Catalan bare-name sentences at a mirror-image 2.5 average. While not unlikely, this is a weaker expectation in the direction of Catalan to Spanish, since formal descriptions of standard Catalan grammars already describe a language in permanent contact with Spanish, whereas standard descriptions of Spanish are rarely of contact varieties. In other words, our expectation of (very) low ratings for bare name sentences in Catalan is created by descriptions that already account for contact with Spanish (i.e., there is no no-contact Catalan baseline to compare this to).

Besides this bidirectional influence, there is an alternative/complementary sociolinguistic explanation. Articles in front of (some) first names are attested in some registers of Spanish, although as a stigmatized feature systematically censured in prescriptive grammars, the school system, and the media [67, §13.5.6]. To the extent that native grammatical features survive the filters of literacy and sociolinguistic prescriptivism, and since features from all registers familiar to a speaker are likely to influence their judgements even if they would not surface in their production, it is not completely unexpected to see Det+Name sentences being rated above the bare minimum at times, although the expected preference for bare names remains visible in the ceiling ratings for constructions without the determiner.

### 7.2. Word order

The results of the Word order condition in Catalan and Spanish are not *qualitatively* unexpected, but they are *quantitatively* different from potential expectations created by standard descriptions. Our participants show no preference in Spanish between VOS and VSO, whereas they significantly prefer the former in Catalan. This pattern is replicated in English, where VOS sentences receive significantly higher ratings than VSO ones. The interaction analyses confirm a similar effect size in both languages, with a main effect of Subcondition in the absence of an interaction with Language in the English-Catalan combined data set.

However, the ratings are somewhat unexpected in quantitative terms. Both marked word orders receive a relatively low score even in Spanish, where the grammar licenses both under the right conditions—and the same is true of VOS in Catalan. This may have to do with the fact that the AJT was in the written modality, presented as single sentences outside of any discourse context. In the absence of a pragmatic context to induce a relevant marked word order and/or associated prosodic cues in Spanish, our participants might have had problems accommodating the non-canonical orders within their associated information structure. Therefore, the relatively low scores for these subconditions might reflect their preference for an unmarked word order in the absence of relevant cues. Importantly, however, the predicted differences between subconditions survived the low absolute ratings in both Spanish and Catalan: VSO and VOS do not differ significantly in Spanish, and VOS is still significantly preferred to VSO in Catalan (and English).

### 7.3. Differential object marking

At first sight, and considering traditional descriptions of standard Catalan grammars, results for the DOM sentences in English could have been taken to evidence transfer from Catalan, whereby sentences with DOM are rated as considerably worse than their counterparts without DOM. This happened irrespective of whether the speakers were dominant in Catalan or Spanish. Somewhat unexpectedly, however, in Catalan our participants provided equally high ratings to sentences with and without DOM (see also [68, 69] for similar results, and for a discussion of online vs. offline measures). The interaction analyses, although lacking a Subcondition by Language interaction in the English-Catalan combined data set, confirmed that DOM was rated significantly lower only in English. The data in the English-Spanish analyses were clearer: the effects are of opposite sign.

The high ratings in Catalan overall suggest that, in contrast to Spanish, the Catalan grammar of these speakers does not associate a semantic or syntactic effect to DOM and, in essence, that the presence of DOM is optional in their Catalan. Catalan does not exhibit the properties expected from varieties with syntactic DOM [70], such as an extension of dative clitics to accusative contexts when the (human, masculine) DP receives DOM—something that happens across several Spanish varieties.

(10) a. Vi     a Pedro.          *Spanish*

     saw.1sg DOM Pedro

     ‘I saw Pedro’

     b. Lo/Le     vi.

     him.acc/him.dat saw.1sg

     ‘I saw him (dative)’

We propose that the surface optionality of DOM in Catalan is an instance of Labovian variation, meaning that Catalan DOM is not a syntactic feature but an instance of optional case marking at Phonological Form (PF). The equally high rates in Catalan support this analysis for three reasons: (i) DOM seems to have no syntactic or semantic effects, as the ratings are the same in both sets of sentences; (ii) it is optional for one and the same speaker, who assigns equally high grades to sentences with and without DOM, and (iii) it may reflect social or contextual properties outside of grammar, such as style, the identity of the speaker or the type of personal relation with the interlocutor, because DOM is condemned in prescriptive grammars and thus associated to less formal registers. Following Nevins and Parrott [71], who analyze Labovian variation as the effect of probabilistic PF rules that apply in some contexts, we propose that Catalan DOM obtains from the application of a probabilistic rule that introduces the exponent *a* to mark case (cf. Noyer’s [72] notion of dissociated morpheme) in DPs that are syntactically assigned accusative case, as in (11).

(11) ø (_0 <_ *p*_*a*_ < _1)_ ⟶ a / _____ DP_[accusative]_

The most important fact to take away from this discussion is that, if on the right track, an optional PF rule would account for the high acceptance of DOM sentences in the Catalan data without the need to assume the underlying syntax for this property, which would conflict with other facts about the language. This means that even if the Catalan grammar was transferred as the basis for the initial L3 grammar of English, this would not include the underlying syntax of a DOM property.

As an anonymous reviewer points out, one may wonder why then we should not expect this optionality at PF to be present in English. In our view, if this Labovian type of optionality is strictly speaking not part of the grammar, it finds no need to be mapped onto a form in English that is non-overlapping. Things might be a little different in the comparison between Spanish and Catalan, where the morphophonological form (‘*a*’) exists in Catalan for independent reasons/functions that overlap with analogous functions in Spanish (e.g., dative marking).

### 7.4. Causative constructions

Data from the causative constructions are by far the most complex. The Spanish and Catalan results did not meet our initial expectations here either, nor did the English data transparently map to the Catalan one in terms of patterns of difference between subconditions. Taken at face value, these results do not support the predictions of the TPM, although they are equally problematic for alternative hypotheses—for example, that they should follow the patterns of the Spanish data. Similarly, it is unclear whether and how hybrid influence from both languages [e.g., 10] could be read from these results, or what conditioning variables would have weighted the relative influence of each language. In the clitic condition, participants rated English sentences significantly higher than expected, by any account—recall that the order causative-clitic-infinitive is ungrammatical in both Spanish and Catalan. Additionally, English sentences in the DP condition received significantly lower ratings than their causative+(clitic) pronoun counterparts, differently from the results in the Catalan AJT. Interaction analyses showed main effects of Subcondition and Language as well as their interaction in both the English-Catalan and Spanish-Catalan combined data sets, and evidenced a similar distance between each previous language and English, with the same contrast between C+Pro and C+DP taking an opposite sign in English.

We propose a learning-related explanation whereby these results can be (somewhat better) understood if our participants did not interpret the English pronouns as clitics, in contrast with their immediate equivalents in Spanish and Catalan. The distribution of English pronouns does not correspond to the behavior of a Romance clitic pronoun in very salient and frequent surface properties. For instance, English personal pronouns can stand alone as answers to questions, where they are focalized (12), while their Spanish and Catalan translations cannot, as expected from clitics (13).

(12)    – To whom did you speak?                   *English*

    – Him.

(13)    a.    – ¿A quién hablaste?                   *Spanish*

    To who *pro* speak._2nd/sing.past_

    – {*Le / A él}

    him / to he

b.    – Amb qui vas parlar?                   *Catalan*

    With who *pro* PFTV._2nd/sing.past_ speak

    – {*li / amb ell}

him / with he

Given the saliency of such contrasts and the ubiquity of personal pronouns (which learners are likely to come across in almost every English sentence), it is likely that our Catalan-Spanish bilinguals would realize very early on that their pronouns are not syntactically equivalent to the ones in English sentences. This could explain why our participants rate English sentences seemingly independently of their either previous grammar: if the Romance clitics cannot be mapped onto the English pronouns, the sentences become non-equivalent, with cascading effects for these causative structures.

If an *English strong pronoun ≠ Romance clitic* distinction is in place, the principle underlying our results might be related to the syntactic size of the argument. Following Cardinaletti and Starke [73], we assume that DPs, strong pronouns and clitics differ in structural complexity. Clitics, the relevant pronouns in Spanish and Catalan, involve the least functional structure; strong pronouns—as in English—are more syntactically complex; and DPs are full-fledged structures. On this assumption, what Spanish and Catalan do is to reanalyze the infinitive with the causative verb across a clitic pronoun, which introduces minimal syntactic structure:

(14) Causative–clitic–infinitive ⟶ [causative + infinitive]–clitic

Because the English pronoun is a strong one, it introduces more structure between the causative and the infinitive, making the reanalysis in (14) impossible. In this context, transfer from Catalan into English is compatible with the C+Pro results if speakers have already learned that the pronoun is syntactically more complex than a clitic, because that would be enough to block the reanalysis:

(15) Causative–strong pronoun–infinitive ⟶ *[causative + infinitive]–strong pronoun

If the reanalysis is blocked by the strong pronoun, this will also be true of a DP, which is even more complex. This would explain why our participants also assign higher rates to English sentences where the DP is between the two verbs.

(16) Causative–DP–infinitive ⟶ *[causative + infinitive]–DP

In summary, we propose that results from the Causative condition are unexpected at first sight from any L3/L*n* transfer model, but they might not be uninterpretable altogether. If treating English pronouns as different from Catalan and Spanish clitics forced our speakers to postulate an additional rule to analyze the English sentences, these data seem less randomly distributed. This is particularly interesting because it illustrates unavoidable confounds that might be introduced by the natural course of language acquisition operating over potentially transferred initial representations. While testing *ab initio* learners and controlling the input they have access to is a step in the right direction, one cannot preclude (nor would want to for reasons of ecological validity) learning/acquisition over the course of the two month program, for at least some properties. This will add some noise to the transfer “signal”.

## 8. General discussion

We set out to investigate two research questions. The first was whether testing multiple (unrelated) domains of grammar across all three languages of beginner L3 learners would yield evidence in support of either full or property-by-property transfer. The second was whether these data would favor any current L3 transfer model over others. Similar performance in L3 English and one of the previous languages throughout would constitute strong evidence of full transfer. It is not clear to us that the opposite is true. Dissimilar performance between the L3 and the L1 and/or L2 would not constitute strong evidence for property-by-property transfer, because it could simply stem from unsystematic influence. Instead, property-by-property transfer would be best supported by L3 performance that resembles the L1 or the L2 depending on the property, if the data conforms to some sort of systematicity hypothesized beforehand.

We derived the TPM’s predictions for this language combination. These reflect the expectation that Catalan be selected as the transfer source for the initial L3 English grammar, based on its larger phonological proximity to English. Starting out with an L3 grammar for English that is essentially a copy of their Catalan grammar should result in similar judgements between these languages across the board, to the extent that we capture these speakers’ L3 before that first grammar starts to change. Alternatively, the TPM might be right in assuming full transfer, but wrong in predicting Catalan over Spanish. Neither of these predictions is directly supported, since we did not find an obvious alignment *in all conditions* between the English judgements of our subjects, on the one hand, and the ones they provided for the same conditions in Catalan and Spanish, respectively. These facts are equally problematic for default models (as behavior was independent of order of acquisition) or the CEM, which precludes non-facilitative transfer (exemplified most clearly in our data by the preference for Det+Name constructions in English).

Another possibility, in line with models such as the LPM, is that properties are sampled independently from each previous language. However, the English results in some conditions (e.g., Causatives) do not clearly point to either Catalan or Spanish, so a first-pass evaluation of this prediction does not bear out either. At this point, we must acknowledge that our results do not squarely support any available theory. In section 7, we offered an interpretation of what might be going on in our data. We took as a guide the more explicit set of predictions, those of the TPM, and evaluated their compatibility with our data. This meant determining whether our English results in each condition are understandable assuming a Catalan-like representation, or alternatively can be traced back to a previous state of the grammar when this was the case.

Overall, we believe that our interpretations in section 7 above come together to support the idea that these speakers’ first L3 English grammar was transferred from Catalan. From that starting point, different independent processes may have produced the patterns of data we observe. Initial transfer from Catalan is further (and more unambiguously) supported by data from two other domains of grammar tested in these same learners and reported elsewhere (definiteness effects in [11], and Negative Polarity items in [9]), as well as by data from other domains in different speakers of the same population [26].

In our view, property-by-property transfer is inconsistent with these data on at least two fronts. First, the proficiency of our participants was very low at the point of testing, which makes it unlikely that transfer for the more complex properties should have happened yet, if one defends a developmentally moderated account (transfer when needed). Transferring only when needed would help the learner to avoid precipitous instances of transfer from a less ideal source, which would have to be unlearned over the course of development. From this view, it follows that an early L3 grammar should be mostly underspecified (i.e., ambivalent with respect to most grammatical properties), and judgements should therefore present a high degree of inconsistency and fluctuation, especially when Catalan and Spanish are at odds. While this is variable in our data set, we believe that the comparable consistency in the judgements of our participants across the three languages is a sign of specification for the relevant properties.

The second inconsistency between property-by-property transfer and our results is that some of them, if they indeed reflect transfer from Catalan, would be instances of non-facilitative transfer. While modern accounts of this kind do allow for this to happen in principle, they still do not offer predictions anticipating this type of outcome, especially when transferring from the other grammar would have been beneficial.

A number of limitations should be acknowledged. First, our sample size (N = 40) is small for the number of factors we are seeking to model, especially considering that our analyses include an interaction with a non-categorical variable (dominance), which raises the number of required observations. While our sample is comparatively large in the context of L3 studies, future research should aim at examining larger cohorts of speakers, if there is sufficient availability. If available groups are inevitably small (a typical situation in naturalistic L3 studies), then this should be compensated with a higher number of items. Westfall, Kenny and Judd [74] offer some practical guidance to conduct power analyses that consider the number of items alongside the number of participants. Depending on the estimated size of the effect, substantially increasing the number of items for a relatively small sample size might help overcome power limitations. In our case, a higher number of items (20 per condition) would have avoided the potential inflation of Type I and Type II errors that low power brings about, as the total number of observations per cell in our study falls short of the recommendations in Westfall et al.

The second limitation has to do with the nature of the evidence brought to bear on the L3 theoretical debates presented here. As with most research on linguistic transfer, the patterns presented here are ultimately correlational: we merely note that behavior in language X matches or mismatches behavior in language Y. Deriving causal arguments from correlational data can of course be problematic, since what we attribute to influence from a previously acquired language may (also) be due to other factors. Given the sheer number of variables involved in language development, it is difficult to say what constitutes unambiguous causal proof. Controlling potential nuisance variables arguably adds ‘causal weight’ to correlations, because it reduces the number of competing causal accounts. We believe that our method has some advantages in this respect. By testing very close to first exposure and controlling (effectively limiting) the exact amount and quality of L3 input the learners received, we reduce the explanatory power of variables related to input/exposure. For example, it is less likely that our participants’ L3 judgements reflect ‘learner effects’ common to all English learners regardless of prior experience [75]. Thus, by reducing the likelihood of input playing a significant role, chances increase for the L3 data to reflect the influence of previous languages more cleanly. Nevertheless, we are conscious of this inherent limitation, and are cautious in interpreting our results.

Our data do not directly speak to the intricacies of L3/L*n* development, but we maintain that determining the starting point of the L3 journey is the only way to maximize our chances of faithfully mapping the entire route. However, the initial conditions of L3 acquisition only constrain the trajectory in some ways: they do not ultimately determine the range of outcomes of the process. There is no reason to expect that, over time and regardless of what the first L3 grammar looked like, L3 learning cannot be successful. Despite two decades of work, it is still early days in L3/L*n* transfer studies, and so it is likely that none of the current theories are correct in absolute terms. The TPM is no exception, but we believe that the data from this study are compatible with its predictions overall or, at the very least, do not qualify as strong enough evidence to preclude full transfer as a viable option in L3 acquisition.

## Supporting information

S1 File Full list of stimuli. (DOCX)

S2 File Model formulas and full output tables. (DOCX)
