# Supplementary material for: On the status of transfer in adult third language acquisition of early bilinguals
Source: PLoS One. 2021 Mar 4;16(3):e0247976. doi: 10.1371/journal.pone.0247976 (PMC7932084; doi:10.1371/journal.pone.0247976)
Supplement: S1 File — (DOCX) [file pone.0247976.s001.docx]

| **S1 File.** Full list of stimuli | | |
| --- | --- | --- |
| **Table S1.1.** English Stimuli | | |
| DOM | No DOM | My mother invited my friend. |
|  |  | My friend hugs his boss every day. |
|  |  | The politician impressed the boy in the room. |
|  |  | the student sees the English teacher every morning. |
|  |  | the doctor helped that girl. |
|  | DOM | The teacher offended to his student. |
|  |  | The child insulted to the teacher in the room. |
|  |  | The teacher hugs to the teacher every day. |
|  |  | The dog attacked to this boy. |
|  |  | The girl impressed to her admirer. |
| Det + Name | Det + Name | The doctor is talking to the Mary. |
|  |  | The teacher talked to the Michael. |
|  |  | The teacher is dancing with the Samantha. |
|  |  | The student talked to the Sam about the exam. |
|  |  | The waiter is dancing with the Martha. |
|  | Name | The director is talking to James about the exam. |
|  |  | The doctor is dancing with Peter. |
|  |  | The teacher is talking to Tom. |
|  |  | Call me if the girl talks to John about the book. |
|  |  | The doctor talked to Michael. |
| Word Order | VOS | Will buy a book the girl. |
|  |  | Draws a picture the girl. |
|  |  | Eats an apple the boy. |
|  |  | Drinks coffee the student. |
|  |  | Drinks tea the doctor. |
|  | VSO | Will play the boy football. |
|  |  | Will bring the boy a ball. |
|  |  | Will walk the girl to the park. |
|  |  | Eats the girl pizza. |
|  |  | Drinks the boy a beer. |
| Causatives | C + Pro | The girl made him buy an apple. |
|  |  | The boy made him eat an apple. |
|  |  | The doctor made him eat vegetables. |
|  |  | The boy made him pay the coffee. |
|  |  | The girl made him wear a black T-shirt. |
|  | Per + Pro | The teacher is making him write a book. |
|  |  | The girl is making him read a book. |
|  |  | The teacher is making him do homework. |
|  |  | The doctor is making him write a book. |
|  |  | The teacher is making him stop smoking. |
|  | C + DP | The boy made the girl read a book. |
|  |  | The girl made the teacher answer the question. |
|  |  | The teacher made the boy read the sentences. |
|  |  | The teacher made the boy do homework. |
|  |  | My friend made the girl drink coffee. |

| **Table S1.2.** Catalan Stimuli | | |
| --- | --- | --- |
| DOM | No DOM | La professora va veure la seva millor alumna. |
|  |  | El policia va arrestar la presidenta d'Alemanya. |
|  |  | El periodista va entrevistar la dona més rica de Barcelona. |
|  |  | El mestre va renyar aquesta nena. |
|  | DOM | La professora va abraçar a la meva mare. |
|  |  | La meva amiga va conèixer a la meva mare. |
|  |  | El polític va insultar a aquella periodista. |
|  |  | El metge va curar a aquest noi. |
| Det + Name | Det + Name | La mestra va barallar-se amb la Marta. |
|  |  | La Carlota anirà a la platja demà. |
|  |  | La Laura acabarà els deures abans de classe. |
|  |  | El periodista va parlar amb la Laia del seu nou llibre. |
|  | Name | Marta anirà a la platja demà passat. |
|  |  | El professor va quedar amb els pares de Cristina. |
|  |  | Anna volia un gelat ahir a la tarda. |
|  |  | Maria va dir que no vindria a la festa. |
| Word Order | VOS | Van demanar una cervesa els nois. |
|  |  | Comprarà un ordinador el professor. |
|  |  | Beu cafè per esmorzar la noia. |
|  |  | Està escrivint un llibre el professor. |
|  | VSO | Ha portat el noi un regal. |
|  |  | Han comprat els nois uns llibres. |
|  |  | Portarà la noia una brusa blava. |
|  |  | Va saber el nen la resposta. |
| Causatives | C + Pro | La noia farà li portar una brusa negra. |
|  |  | El professor farà li acabar els deures. |
|  |  | La seva mare farà li comprar un mòbil nou. |
|  |  | El noi farà li arribar tard a la reunió. |
|  | Per + Pro | La noia va fer-li publicar un llibre. |
|  |  | La noia va fer-li escriure una carta al director. |
|  |  | La monitora va fer-li beure un got de llet. |
|  |  | El periodista va fer-li dir la veritat. |
|  | C + DP | L'encarregat va fer la noia obrir la porta. |
|  |  | La professora farà els nens acabar l'exercici. |
|  |  | El metge farà el pacient menjar més verdura. |
|  |  | El noi va fer la noia recollir els papers de terra. |

| **Table S1.3.** Spanish Stimuli | | |
| --- | --- | --- |
| DOM | No DOM | La mujer ofendió este policía. |
|  |  | El chico impresionó estas chicas. |
|  |  | El médico curó ese deportista. |
|  |  | La muchacha abrazó el protagonista de la película. |
|  | DOM | El periodista entrevistó a este político hace tiempo. |
|  |  | La chica atacó a ese niño. |
|  |  | El médico ayudó a estas chicas. |
|  |  | El chico insultó a ese policía. |
| Det + Name | Det + Name | La enfermera se casó con el Pablo. |
|  |  | El Jesús trajo el champán. |
|  |  | Estos bombones son para la Sandra. |
|  |  | Ayer vino el Borja. |
|  | Name | El Policía discutió con Paula. |
|  |  | Rosario se olvidó las cervezas. |
|  |  | Este paquete es para Miguel. |
|  |  | Anoche llegó Susana. |
| Word Order | VOS | Ha comprado una cerveza tu hermana. |
|  |  | Enviaron el paquete mis hermanos. |
|  |  | Vende su coche la chica. |
|  |  | Está leyendo un libro el profesor. |
|  | VSO | Ha traído el chico las flores. |
|  |  | Está escribiendo mi hermana una carta. |
|  |  | Tiene el profesor un regalo para mi madre. |
|  |  | Entregó Miguel un paquete. |
| Causatives | C + Pro | Su mujer hizo le comprar este coche. |
|  |  | El camarero hizo lo probar su postre. |
|  |  | La enfermera hizo lo quitarse la venda. |
|  |  | El taxista hizo le pagar el doble. |
|  | Per + Pro | El profesor está haciéndole publicar su novela. |
|  |  | El médico va a hacerle dejar el tabaco. |
|  |  | La mujer va a hacerle pagar la cena. |
|  |  | El periodista está haciéndole lamentar su decisión. |
|  | C + DP | Mi amigo hizo al pintor vender los cuadros. |
|  |  | Mi hermana hizo al camarero traer el postre. |
|  |  | La chica hizo al político responder las preguntas. |
|  |  | El detective hizo al acusado confesar el crimen. |
