# Supplementary material for: On the status of transfer in adult third language acquisition of early bilinguals
Source: PLoS One. 2021 Mar 4;16(3):e0247976. doi: 10.1371/journal.pone.0247976 (PMC7932084; doi:10.1371/journal.pone.0247976)
Supplement: S2 File — (DOCX) [file pone.0247976.s002.docx]

**S2 File.** Model formulas and full output tables.

This appendix lists the formulas and fixed effects estimates for the ordinal mixed regression models used to analyze each subset of the data. Note that the formulas use *R* notation, where asterisks represent all main effects and interactions of the factors involved, and the random terms are included within parentheses, with “1|factor” representing a random intercept for *factor1*, and “factor1|factor2” indicating a random intercept for *factor2* plus a correlated random slope for *factor 1* within *factor2*. Note that, in the outcome of an ordinal mixed regression, (the provided) threshold coefficients correspond to the intercepts in logistic regressions. Estimates are provided in log odds ratios. The reference subcondition, or level, is indicated for each dataset. Rows pertaining to significant factors or interactions (other than the intercept) are highlighted. Threshold coefficients are provided below the fixed effects (*p*-values are not provided in the *clmm* outcome).

**S2.1. Determiner + Name**

DET+NAME (i.e., sentences with a determiner preceding the proper noun) was set as the reference subcondition for all datasets on the Determiner + Name data.

**S2.1.1. Catalan**

Final model:

response ~ level*dominance + (1|participant) + (1||item)

**Table S2.1.** Model estimates, standard errors, *z*- and *p*-values for the fixed factors, and threshold coefficients in the final model for the Determiner + Name data in Catalan.

| Factor | Estimate | S. Error | z value | *p* |
| --- | --- | --- | --- | --- |
| Level | -4.36 | 0.46 | -9.44 | < .001 |
| Dominance | -0.53 | 1.72 | -0.31 | .76 |
| Level by Dominance | 0.30 | 1.63 | 0.18 | .86 |
| 1\|2 | -5.35 | 0.54 | -9.99 |  |
| 2\|3 | -4.22 | 0.48 | -8.76 |  |
| 3\|4 | -3.03 | 0.42 | -7.26 |  |

**S2.1.2. Spanish**

Final model:

response ~ level*dominance + (1||participant) + (1|item)

**Table S2.2.** Model estimates, standard errors, *z*- and *p*-values for the fixed factors, and threshold coefficients in the final model for the Determiner + Name data in Spanish.

| Factor | Estimate | S. Error | z value | *p* |
| --- | --- | --- | --- | --- |
| Level | 3.41 | 0.36 | 9.40 | < .001 |
| Dominance | 1.69 | 0.74 | 2.30 | < .001 |
| Level by Dominance | -0.98 | 1.23 | -0.80 | .43 |
| 1\|2 | -1.20 | 0.22 | -5.58 |  |
| 2\|3 | -0.08 | 0.19 | -0.42 |  |
| 3\|4 | 1.25 | 0.22 | 5.78 |  |

**S2.1.3. English**

Final model:

response ~ level*dominance + (1|participant) + (1|item)

**Table S2.3.** Model estimates, standard errors, *z*- and *p*-values for the fixed factors, and threshold coefficients in the final model for the Determiner + Name data in English.

| Factor | Estimate | S. Error | z value | *p* |
| --- | --- | --- | --- | --- |
| Level | -0.72 | 0.19 | -3.71 | < .001 |
| Dominance | -0.62 | 0.56 | -1.11 | .27 |
| Level by Dominance | -0.28 | 0.80 | -0.35 | .73 |
| 1\|2 | -1.19 | 0.18 | -10.73 |  |
| 2\|3 | -0.53 | 0.15 | -3.66 |  |
| 3\|4 | 0.62 | 0.15 | 4.23 |  |

**S2.2. Word order**

VOS word order was set as the reference subcondition for all datasets on the Word order data.

**S2.2.1. Catalan**

Final model:

response ~ level*dominance + (dominance + level:dominance|participant) + (1|item)

**Table S2.4.** Model estimates, standard errors, *z*- and *p*-values for the fixed factors, and threshold coefficients in the final model for the Word order data in Catalan.

| Factor | Estimate | S. Error | z value | *p* |
| --- | --- | --- | --- | --- |
| Level | -1.22 | 0.34 | -3.58 | < .001 |
| Dominance | 0.35 | 1.59 | 0.22 | .82 |
| Level by Dominance | 0.44 | 2.11 | 0.21 | .84 |
| 1\|2 | -0.54 | 0.36 | -1.51 |  |
| 2\|3 | 1.29 | 0.37 | 3.49 |  |
| 3\|4 | 2.30 | 0.40 | 5.78 |  |

**S2.2.2. Spanish**

Final model:

response ~ level*dominance + (1|participnt) + (1|item)

**Table S2.5.** Model estimates, standard errors, *z*- and *p*-values for the fixed factors, and threshold coefficients in the final model for the Word order data in Spanish.

| Factor | Estimate | S. Error | z value | *p* |
| --- | --- | --- | --- | --- |
| Level | -0.33 | 0.23 | -1.41 | .16 |
| Dominance | -0.01 | 0.64 | -0.02 | .99 |
| Level by Dominance | -0.71 | 0.89 | -0.79 | .43 |
| 1\|2 | -1.46 | 0.20 | -7.46 |  |
| 2\|3 | 0.09 | 0.17 | 0.54 |  |
| 3\|4 | 2.39 | 0.25 | 9.5 |  |

**S2.2.3. English**

Final model:

response ~ level*dominance + (1|participant) + (1|item)

**Table S2.6.** Model estimates, standard errors, *z*- and *p*-values for the fixed factors, and threshold coefficients in the final model for the Word order data in English.

| Factor | Estimate | S. Error | z value | *p* |
| --- | --- | --- | --- | --- |
| Level | -0.59 | 0.20 | -2.93 | < .001 |
| Dominance | 0.04 | 0.60 | 0.07 | .94 |
| Level by Dominance | 1.61 | 0.88 | 1.84 | .07 |
| 1\|2 | -0.77 | 0.17 | -4.75 |  |
| 2\|3 | 0.67 | 0.16 | 4.21 |  |
| 3\|4 | 2.02 | 0.21 | 9.70 |  |

**S2.3. Differential Object Marking (DOM)**

No DOM (sentences without DOM) was set as the reference subcondition for all datasets on the Differential Object Marking data.

**S2.3.1. Catalan**

Final model:

response ~ level*dominance + (level+dominance|participant) + (level|item)

**Table S2.7.** Model estimates, standard errors, *z*- and *p*-values for the fixed factors, and threshold coefficients in the final model for the Differential Object Marking data in Catalan.

| Factor | Estimate | S. Error | z value | *p* |
| --- | --- | --- | --- | --- |
| Level | -0.27 | 0.80 | -0.33 | .74 |
| Dominance | 0.34 | 1.83 | 0.19 | .85 |
| Level by Dominance | 3.69 | 2.03 | 1.82 | .07 |
| 1\|2 | -4.40 | 0.70 | -6.28 |  |
| 2\|3 | -3.13 | 0.64 | -4.88 |  |
| 3\|4 | -2.06 | 0.61 | -3.38 |  |

**S2.3.2. Spanish**

Final model:

response ~ level*dominance + (1|participant) + (1|item)

**Table S2.8.** Model estimates, standard errors, *z*- and *p*-values for the fixed factors, and threshold coefficients in the final model for the Differential Object Marking data in Spanish.

| Factor | Estimate | S. Error | z value | *p* |
| --- | --- | --- | --- | --- |
| Level | 3.59 | 0.31 | 11.43 | < .001 |
| Dominance | 0.40 | 0.69 | 0.58 | .565 |
| Level by Dominance | -0.30 | 0.99 | -0.30 | .76 |
| 1\|2 | 0.16 | 0.17 | 0.91 |  |
| 2\|3 | 0.99 | 0.19 | 5.27 |  |
| 3\|4 | 2.76 | 0.27 | 10.26 |  |

**S2.3.3. English**

Final model:

response ~ level*dominance + (1|participant) + (dominance|item)

**Table S2.9.** Model estimates, standard errors, *z*- and *p*-values for the fixed factors, and threshold coefficients in the final model for the Differential Object Marking data in English.

| Factor | Estimate | S. Error | z value | *p* |
| --- | --- | --- | --- | --- |
| Level | -1.09 | 0.21 | -5.31 | < .001 |
| Dominance | -0.29 | 0.68 | -0.42 | .67 |
| Level by Dominance | 0.68 | 0.85 | 0.81 | .42 |
| 1\|2 | -1.84 | 0.20 | -9.32 |  |
| 2\|3 | -0.63 | 0.17 | -3.66 |  |
| 3\|4 | 0.67 | 0.17 | 3.95 |  |

**S2.4. Causative constructions**

C+Pro (sentences with a causative followed by a pronoun) was set as the reference subcondition for all datasets in the Causative constructions data.

**S2.4.1. Catalan**

Final model:

response ~ level*dominance + (level|participant) + (1|item)

**Table S2.10.** Model estimates, standard errors, *z*- and *p*-values for the fixed factors, and threshold coefficients in the final model for the Causative constructions data in Catalan.

| Factor | Estimate | S. Error | z value | *p* |
| --- | --- | --- | --- | --- |
| Level (Per+Pro) | 7.02 | 0.78 | 9.00 | <.001 |
| Level (C+DP) | 0.84 | 0.58 | 1.46 | .14 |
| Dominance | -0.29 | 1.24 | -0.24 | .81 |
| Level (Per+Pro) by Dominance | 4.73 | 2.23 | 2.12 | <.05 |
| Level (C+DP) by Dominance | 0.16 | 1.74 | 0.091 | .93 |
| 1\|2 | 2.60 | 0.39 | 6.62 |  |
| 2\|3 | 4.14 | 0.46 | 9.00 |  |
| 3\|4 | 5.20 | 0.51 | 10.27 |  |

Post hoc comparisons using Tukey Honest Significant Difference (HSD):

**Table S2.11.** Estimates, standard errors, *z-* and *p-*values for the linear hypotheses in the post hoc analysis of the Level main effect in the Catalan Causative constructions data.

| Linear hypothesis | Estimate | S. Error | z value | *p* |
| --- | --- | --- | --- | --- |
| Per+Pro – C+Pro = 0 | 7.025 | 0.78 | 9.00 | < .001 |
| C+DP – C+Pro = 0 | 0.84 | 0.56 | -1.47 | .31 |
| C+DP – Per+Pro = 0 | -6.18 | 0.86 | -7.20 | <.001 |

**S2.4.2. Spanish**

Final model:

response ~ level*dominance + (1|participant) + (1|item)

**Table S2.12.** Model estimates, standard errors, *z*- and *p*-values for the fixed factors, and threshold coefficients in the final model for the Causative constructions data in Spanish.

| Factor | Estimate | S. Error | tzvalue | *p* |
| --- | --- | --- | --- | --- |
| Level (Per+Pro) | 8.29 | 0.82 | 10.09 | <.001 |
| Level (C+DP) | 3.63 | 0. | 5.20 | <.001 |
| Dominance | 1.90 | 1.86 | 1.02 | .31 |
| Level (Per+Pro) by Dominance | -3.88 | 2.14 | -1.81 | .07 |
| Level (C+DP) by Dominance | -2.89 | 1.94 | -1.49 | .14 |
| 1\|2 | 3.01 | 0.55 | 5.44 |  |
| 2\|3 | 4.33 | 0.58 | 7.45 |  |
| 3\|4 | 6.27 | 0.63 | 9.94 |  |

Post hoc comparisons using Tukey Honest Significant Difference (HSD):

**Table S2.13.** Estimates, standard errors, *z-* and *p-*values for the linear hypotheses in the post hoc analysis of the Level main effect in the Spanish Causative constructions data.

| Linear hypothesis | Estimate | S. Error | z value | *p* |
| --- | --- | --- | --- | --- |
| Per+Pro – C+Pro = 0 | 8.30 | 0.82 | -10.09 | < .001 |
| C+DP – C+Pro = 0 | -3.63 | 0.70 | -5.20 | < .001 |
| C+DP – Per+Pro = 0 | -4.67 | 0.70 | -6.67 | <.001 |

**S2.4.3. English**

Final model:

response ~ level*dominance + (dominance|participant) + (1|item)

**Table S2.14.** Model estimates, standard errors, *z*- and *p*-values for the fixed factors, and threshold coefficients in the final model for the Causative constructions data in English.

| Factor | Estimate | S. Error | z value | *p* |
| --- | --- | --- | --- | --- |
| Level (Per+Pro) | 0.79 | 0.20 | 3.93 | <.001 |
| Level (C+DP) | -0.88 | 0.21 | -4.28 | <.001 |
| Dominance | -0.96 | 0.73 | -1.32 | .19 |
| Level (Per+Pro) by Dominance | -0.21 | 0.85 | -0.24 | .81 |
| Level (C+DP) by Dominance | 0.02 | 0.88 | 0.02 | .99 |
| 1\|2 | -1.16 | 0.17 | -6.85 |  |
| 2\|3 | 0.37 | 0.16 | 2.29 |  |
| 3\|4 | 1.62 | 0.18 | 9.06 |  |

Post hoc comparisons using Tukey Honest Significant Difference (HSD):

**Table S2.15.** Estimates, standard errors, *z-* and *p-*values for the linear hypotheses in the post hoc analysis of the Level main effect in the English Causative constructions data.

| Linear hypothesis | Estimate | S. Error | z value | *p* |
| --- | --- | --- | --- | --- |
| Per+Pro – C+Pro = 0 | 0.79 | 0.20 | 3.94 | < .001 |
| C+DP – C+Pro = 0 | 0.88 | 0.21 | -4.27 | < .001 |
| C+DP – Per+Pro = 0 | -1.67 | 0.21 | -7.80 | <.001 |
